# Supplementary material for: Time to recovery and predictors of severe community-acquired pneumonia among pediatric patients in Debre Markos referral hospital, North West Ethiopia: A retrospective follow-up study
Source: PLoS One. 2020 Sep 25;15(9):e0239655. doi: 10.1371/journal.pone.0239655 (PMC7518609; doi:10.1371/journal.pone.0239655)
Supplement: Attachment — Submitted filename: Response to Reviewers.docx [file pone.0239655.s002.docx]

**Response to Reviewers**

Per the given comments and recommendations, this is a separate file of response to reviewers which addresses comments and suggestions of the reviewers and academic editor.

**Comments to the Author**

1. Is the manuscript technically sound, and do the data support the conclusions?
The manuscript must describe a technically sound piece of scientific research with data that supports the conclusions. Experiments must have been conducted rigorously, with appropriate controls, replication, and sample sizes. The conclusions must be drawn appropriately based on the data presented.

Reviewer #1: Partly

Reviewer #2: Partly

**Response:** Data used for this study is uploaded as separate file in STATA statistical software format as “supporting information”. Conclusion has been made based on the finding of the study.

2. Has the statistical analysis been performed appropriately and rigorously?
Reviewer #1: Yes

Reviewer #2: No

**Response:** statistical analysis is corrected in the revised manuscript per the comments provided. See the method section under analysis (p 8-9).

3. Have the authors made all data underlying the findings in their manuscript fully available?

The [PLOS Data policy](http://www.plosone.org/static/policies.action#sharing) requires authors to make all data underlying the findings described in their manuscript fully available without restriction, with rare exception (please refer to the Data Availability Statement in the manuscript PDF file). The data should be provided as part of the manuscript or its supporting information, or deposited to a public repository. For example, in addition to summary statistics, the data points behind means, medians and variance measures should be available. If there are restrictions on publicly sharing data—e.g. participant privacy or use of data from a third party—those must be specified.

Reviewer #1: No

Reviewer #2: No

**Response:** Data used for this study is uploaded as separate file in STATA version 14 statistical software format as “supporting information”.

4. Is the manuscript presented in an intelligible fashion and written in standard English?
PLOS ONE does not copyedit accepted manuscripts, so the language in submitted articles must be clear, correct, and unambiguous. Any typographical or grammatical errors should be corrected at revision, so please note any specific errors here.
Reviewer #1: No

Reviewer #2: Yes

**Response:** we improved and corrected English grammar, punctuation and spellings in the revised manuscript.

5. Review Comments to the Author

Reviewer #1: This is a retrospective review of children admitted to an Ethiopian hospital with severe community acquired pneumonia. It has regional relevance and potential relevance to other countries with a similar socio-economic profile. I have several suggestions to further improve the article's content and external generalisability.

**Major comments:**

The article must include a better description of context and standard of care, as well as available resources. What are the average numbers of paediatric admissions per annum, and the overall mortality rate? Is PICU available, what level of respiratory/ventilatory support is available on the ward or in the hospital/region (e.g low/high flow oxygen therapy; non-invasive ventilation (CPAP/BiPAP); or invasive ventilation)? What proportion of children received any respiratory support? What antibiotic approach is standardly employed in terms of empiric treatment, and antibiotic stewardship – again, I presume the WHO guidelines were employed, but this should be stated. The only information in this regard is that just over half the children in the study received penicillin. What treatment did the remainder receive?

**Response**: The hospital services for pediatric patients are stated in the revised manuscript under method section (study area) p-3 and result section p-11.

The hospital provides services for children less than 15 years in a separate ward of pediatrics following the national guideline which was adapted from WHO. The hospital received 3293 children per year in average with the available 31 beds. In this hospital, children with severe community acquired pneumonia were admitted in pediatric ward and further diagnose and treatment were provided by pediatricians, general practitioners and nurses. There is no pediatric ICU in the hospital; emergency pediatric conditions were treated in the ward. Diagnosis was made mainly based on clinical findings; Oxygen saturation was recorded and chest X-ray was conducted for patients who had strong indications. Aetiologic micro-organisms of SCAP (bacteria or virus) were not identified. The median time to death for those who died was 6 days . Patients were treated with crystalline pencillin, ceftriaxon, ampicillin and gentamicin based the treatment guidline which is recommended by WHO. Non-invasive respiratory support was given for 14.5% of children who were in respiratory failure (p 10-11).

The above is highly relevant in the discussion section – studies cited include those from Europe, where there is likely to be high levels of intensive care provision, which might explain the longer duration of hospital stay (recovery time), which could be skewed by increased ICU admission and days receiving mechanical ventilation (balanced possibly by lower mortality). In the discussion there is vague reference to “differences in treatment and caring practices, health care settings and other socioeconomic factors between areas where the studies were conducted”. If the setting and healthcare practice at the study site is better described at the outset, then direct reference can be made to the possible reasons for discrepancies in findings.

**Response:** the discussion section of the revised manuscript has been modified as appropriate as possible (p 16-19).

Please provide the case definition of severe CAP - I presume the WHO definition was used, but this should be explicit please. Was any attempt made to differentiate CAP to other LRTI such as bronchiolitis?

**Response:** severe CAP is defined as WHO definition under method section (operational definition p-6). Patients were differentiated from other LRTI clinically and it is stated in the revised manuscript (p-3).

**Other comments:**

Ethics review is indicated as “N/A” on the meta-data form, however in the text it is stated that institutional REC approval was obtained (as appropriate)

**Response:** corrected

Throughout the article there are a number of grammatical errors, and in general the article is quite cumbersome, verbose and difficult to read. I suggest the article undergo language editing before resubmission. There is also a lot of unnecessary repetition of data in tables and text.

**Response:** the article is presented appropriately in understandable manner; English grammar, punctuation and spellings in the revised manuscript are improved. Unnecessary repetition is avoided in the revised manuscript.

What were the organisms associated with CAP – bacterial and viral? Were cultures and viral samples sent for laboratory analysis? Aetiology is important to consider to ensure appropriate antibiotic cover.

**Response:** Aetiologic micro-organisms of SCAP (bacteria or virus) were not identified therefore treatment was given empirically (p-10).

Please list all the symptoms/signs classified as “Danger signs” – were these limited to reduced LOC, abnormal body movement and vomiting? What about hypoxia (SaO2 or presence of cyanosis), convulsions, inability to feed and presence of severe respiratory distress/failure? Was oxygen saturation recorded? Hypoxia on admission has repeatedly been shown to be associated with worse outcome, and monitoring SaO2 is recommended in low resourced countries. I think these data should be presented separately and together, rather than only using the broad category “danger signs” – it is useful to know the relative proportions of children with SCAP presenting with signs of very severe CAP.

**Response:** danger sign is defined in the method section (operational definition p-7) as loss of consciousness, abnormal body movement, vomiting everything, convulsions and inability to feed accompying with Severe CAP. Findings like oxygen saturation <90% or central cyanosis or severe respiratory distress are used to define pneumonia as severe form which inquire hospital admission. Severe CAP is defined as severe respiratory findings and/or danger signs.

**Severe CAP:** pneumonia with any one of: oxygen saturation <90% or central cyanosis or severe respiratory distress or inability to drink or breastfeed or vomiting everything, altered consciousness, and convulsions (p-6).

Patients with pneumonia were classified as mild, moderate and severe in the hospital. Only severe cases were admitted to the hospital for inpatient therapy; of whom this study was conducted. Other patients (mild and moderate) were treated as outpatient cases based on WHO IMNCI guideline. Oxygen saturation was recorded in the hospital as stated in the revised manuscript.

I would like to see acute and chronic comorbid conditions presented separately (can be combined if appropriate for regression analysis).

**Response**: Co-morbidity is defined as any disease condition (acute or chronic) present at admission in addition to SCAP which includes hyperactive airway disease (childhood asthma), retroviral infection, tuberculosis, acute gastroenteritis, pertusis, anemia, meningitis, measles, bronchitis, heart failure and urinary tract infection (p-7). Some children had more than one co morbidity that is why we did not put separately as acute and chronic; difficult to analyze.

Throughout the Results section, please present full data, using n and percent rather than only using percentages. Similarly, where continuous data are presented in the text please present both the central tendency and the data spread (eg IQR).

**Response**: Corrected as recommended.

Table 1 is superfluous, can delete – the data is presented again in Table 3 – just need to add the percentages under the column “Frequency”.

**Response**: Corrected as recommended.

Figure 1 is not clear and probable not needed – rather present the n (%) outcome categories in the text. What outcome/s does the category “others” refer to?

**Response**: outcome is stated in text; figure has removed; no others category from the revised manuscript (explained explicitly). The majority of children (89%) recovered from their illness, 5.11% left against medical advice, 3.69% referred to other institutions and 2.27% have died. (Result section p-10)

P10 - Under the heading “Comparison of Survival Time”, please refer in the text to Tables 3 and 4.

**Response**: corrected as recommended.

Separate headings are given for “Survival Time” and “Time to Recovery”, however these are synonymous (according to case definitions provided). Please stick to one term, to avoid confusion.

**Response**: corrected

P11 – it is not necessary to repeat data presented in the Tables, especially with the lack of precision currently given in the text. Either present all data (median, IQR and significance levels), or simply refer to the tables.

**Response**: unnecessary repetitions are removed in the revised manuscript.
What was the median time to death in those who died?

**Response:** the median time to death was 6 days (p-11).

The statistical analysis sections on pp11-12 I found quite long and difficult to read. I think this could be simplified. Please use full sentences throughout. Eg P12: “Likelihood ratio test which compares log-likelihood statistics for the interaction model and the no-interaction model”. What is this phrase referring to?

**Response:** statistical analysis is modified in method section of the revised manuscript (page 8-9)

The discussion is very long with quite a bit of repetition, and could be shortened without losing important content. Where possible, please refrain from generalizing. On p 16, for example, stating that the finding that children presenting with comorbid conditions recover more slowly is because children with different illnesses take a prolonged time to recover is providing a circular argument for the observed result, it is not explaining the finding at all. Again, if acute and chronic comorbid conditions are reported separately, then more specific arguments can be made, for example related to immunosuppression (HIV infected children). The statement that comorbidity might lead to poor adherence to prescribed drugs leading to prolonged hospitalization is not supported by the study findings, and should be deleted. I also do not see how drugs prescribed and administered in a controlled hospital environment could be affected by “non-adherence”?
**Response**: the discussion section of the revised manuscript is modified (p-16 to19).

There are many more limitations inherent to a retrospective study, which have not been considered here. The one mentioned, that of incomplete medical records, as in fact an initial exclusion criterion, and therefore would not have impacted on the final study findings. The issues of lack of comparators and potential confounders impacting on outcome measures are important potential limitations. For example, when only 31 beds are available for all paediatric admissions, during seasonal increases in other infections (eg gastroenteritis), there may be different admission and discharge criteria, impacting on the “recovery” time point.

**Response**: lack of comparators and failure to include all potential predictors are taken as limitation (p-19). No significant different in admission and discharge criteria in relation to seasonal variation in the hospital.

The conclusion for the first time mentions the BTS guidelines (without citation) – the long time to recovery must be discussed in this regard before the concluding statement.
**Response**: corrected (p-19)

**Reviewer #2:** Pneumonia continues to be a burden and clinical phenotypes may have changed following PCV introduction, hence this is an important topic.
The manuscript test is clear and well-written, but the authors should avoid giving text as bullet points. However, there are quite a few areas where more clarity and rigour over participants, definitions and statistical methods are needed.

Under ‘source population’ it is important to define the diagnostic criteria so that other centres may compare their results. If it is simply the clinician diagnosis, it is important to say if this was determined at the admission or at discharge. However using only the clinician diagnosis would significantly weakening the article.

**Response:** diagnosis was made clinically mainly and investigations like chest x-ray are used. The diagnosis of severe CAP was determined in accordance to WHO (P-10).

Under the sample size section please give the prevalence and effect size that was being targeted in order to justify the sample size calculation.

**Response:** proportions and ratio are explained; effect size (OR/RR) is calculated based on the given data in the software (epi info) so as not needed to state (p-4). The sample size was determined using a double population proportion formula by considering, P1: proportion of exposed (62.3%), P2: proportion of non exposed (47.4%), ratio (1), power (80%), 95% confidence level (p 4-5).

Since time to discharge was used as the primary endpoint, ’length of stay’ or something similar should be used in the title and throughout the text rather than referring to ‘recovery’ since other factors may have altered the hospitalisation duration, especially when severe malnutrition or co-morbidities were present.

**Response:** recovery is defined as children improved from SCAP (p-6). In this study discharge and recovery were not used interchangeably as defined in the method (operational definition section) since children were discharged without improvement due to other reasons like referral and refusal (against medical advice); children discharged when improved or might stay in the hospital for other treatment like malnutrition and co-morbidities although they had improved from SCAP.

Please add to whether or not data on the specific features after severe pneumonia as defined by the World Health Organisation (and the BTS) were available, and why they are not presented in the independent variables. This is important because the signs are used to define with the severity of pneumonia.

**Response:** WHO definition of pneumonia was used (p-6). Severity score of patients with pneumonia is rated as mild, moderate and severe in the hospital where the study was conducted and in Ethiopia in general. Only severe cases are admitted to the hospital for inpatient therapy that is being severe pneumonia is admission criteria by itself. Other patients (mild and moderate) are treated as an outpatient cases. To take danger signs as independent variable in different category, the proportion were not adequate to use.

Among the independent variables please indicate whether continuous values for the anthropometry were used and how they were converted into Z scores.
**Response**: Anthrophometric measurements were compared with WHO child growth standards to classify nutritional status of children (p-10).

For the Cox regression models two methods of variable selection are given bivariate testing of individual variables and a backward stepwise method. Please explain whether these were done in order or whether both were done on the raw data. Please provide the probability for removal criteria used for the backward stepwise selection. Please provide the rationale and the definitions of the strata.

**Response**: bivariable and backward stepwise variable selection methods were used in order with a p-value of 0.25 (p-8)

In the methods please describe how the status of fully vaccinated or partially vaccinated was defined. One problem with this variable is that it is age-dependent and since only children of nine months or more would be eligible for measles vaccine for example.
**Response**: fully vaccination was defined as children who had completed all form of vaccinations; partially vaccinated (children who had taken at least one dose of PCV) and non vaccinated (children who had never vaccinated PCV and other vaccines) p-7

Please also defined terms used in results including hyperactive airways disease and high-grade fever.

**Response**: high grade fever is defined when body temperature becomes >38.^0^c (p-10) and hyperactive airways disease is the national classification of childhood asthma (p-10).
One problem with examining both time to discharge and survival time is that they are competing risks. For example, if a child dies they would not be eligible to have their time to discharge recorded. The usual way to deal with this problem is to undertake competing risks analysis. An example is children with a danger sign, who have a higher risk of mortality for whom the effect on time to discharge could be biased.
**Response**: survival time is the period starting from the date of admission to recovery; for censored observations (discharge without improvement), time is counted for each observation until discharge and used to calculate rate per person time (incidence rate). This is the beauty of survival analysis as it handles such issue. Competing risk could not be a problem because the number of event in this study was high (88.9%) with small proportion of censored observations.

With only 39 events, reporting the risk factors for mortality is likely to be misleading if many independent variables were tested. It would be better to focus on time to discharge.
**Response**: event was operational zed as children recovered from SCAP which were 313 and 39 were censored observations (children who didn’t recover). Here our outcome/independent variable was recovery not mortality; we identified predictors of time to recovery.

Season effects may be important. RSV is typically the leading cause of pneumonia, it is usually highly seasonal. In the tables, some of the 95%Cis are missing. However, it is likely these should be interquartile ranges as time to discharge is skewed (this is correct in the manuscript text). I wasn’t clear what is being tested in the ‘checking no interaction assumption’. If all possible interactions are being tested then this needs to be described in the methods section. The assumption in a Cox model is that effects are not time-varying

**Response**: the robust model of survival analysis is Cox regression (Cox proportional hazard). But in this study proportional hazard assumption was not satisfied as checked by different methods. Therefore extension of the model to stratified Cox regression was needed. The assumption of this model is no interaction assumption which has been checked. These is explained in the revised manuscript as well (page 8-9). Here stratified Cox regression is the extension (advanced forms of) Cox regression in survival analysis.

One point on terminology when reporting the Cox model, the aHR for a feature gives the how much more or less likely a child is to be discharged every day rather than the ‘rate of recovery’.

**Response**: in this study discharge and recovery are not be used interchangeably as defined in the method (operational definition section) since children were discharged without improvement due to other reasons like referral and refusal (against medical advice). In this sense AHR gives how likely each child recovered every day.
