# Supplementary material for: Time to recovery and predictors of severe community-acquired pneumonia among pediatric patients in Debre Markos referral hospital, North West Ethiopia: A retrospective follow-up study
Source: PLoS One. 2020 Sep 25;15(9):e0239655. doi: 10.1371/journal.pone.0239655 (PMC7518609; doi:10.1371/journal.pone.0239655)
Supplement: Attachment — Submitted filename: Review 03326 R1.docx [file pone.0239655.s003.docx]

Thank you for inviting me to review the revision of this manuscript. In general, I find it to be much easier to read and the authors have taken most review comments under consideration. I have one major and minor comments related to this work.

**Major comments**

I am concerned about the outcome of “recovery”, which is highly subjective, being determined simply from clinician assessment. This is likely influences by a range of factors, including bed availability, and may not have been consistent throughout the study period. This should also be considered in the limitation section.

**Minor comments:**

Throughout the manuscript there is still clear evidence that the manuscript has not been edited by a native English speaker. Examples are given below, but after page 7 I stopped looking at grammar, as I believe this is beyond the scope of scientific review.

P3: “The hospital received 3293 children per year in average with the available 31 beds” change to “:The hospital admits an average of 3293 children per year, with 31 available beds”.

P3: “children with severe community acquired pneumonia were admitted in pediatric ward and further diagnose and treatment were provided by pediatricians, general practitioners and nurses” – change to “children with severe community acquired pneumonia

were admitted in**to the** pediatric ward and further **diagnosis** and treatment were provided by

pediatricians, general practitioners and nurses”.

P5: “were admitted by sever community acquired pneumonia” – should be “admitted **with** **severe** CAP”

P5 – the medical charts were not extracted, presumably data were extracted from the medical charts.

P6 - “…inability to feed accompanying with SCAP” – change to “…inability to feed **in addition to** SCAP”

P7 – “fully vaccination was defined…” should be “full vaccination status was defined”

P7 – Very clumsy paragraph: “The patient records were first observed and an appropriate data extraction format was prepared in English. Data collectors used the data collection tool to collect the information from children’s charts. Charts were retrieved using the children’s registration number which was found in the database in the electronic system and the data clerks of the hospital were also support by identifying the charts”.

This can be rewritten much more clearly and simply, for example: “Patient records were retrieved using their unique registration number identified in the electronic hospital database. Charts and records were reviewed and data were extracted using a standardised English data extraction form”.
