# Supplementary material for: Time to recovery and predictors of severe community-acquired pneumonia among pediatric patients in Debre Markos referral hospital, North West Ethiopia: A retrospective follow-up study
Source: PLoS One. 2020 Sep 25;15(9):e0239655. doi: 10.1371/journal.pone.0239655 (PMC7518609; doi:10.1371/journal.pone.0239655)
Supplement: Attachment — Submitted filename: Response to Reviewers.docx [file pone.0239655.s004.docx]

**Response to Reviewers**

We would like thank you for your constructive comments. This is our response that addresses comments and suggestions of the reviewers and academic editor.

Reviewers' comments:

Reviewer's Responses to Questions

**Comments to the Author**

1. If the authors have adequately addressed your comments raised in a previous round of review and you feel that this manuscript is now acceptable for publication, you may indicate that here to bypass the “Comments to the Author” section, enter your conflict of interest statement in the “Confidential to Editor” section, and submit your "Accept" recommendation.

Reviewer #1: (No Response)

Reviewer #2: (No Response)

2. Is the manuscript technically sound, and do the data support the conclusions?

The manuscript must describe a technically sound piece of scientific research with data that supports the conclusions. Experiments must have been conducted rigorously, with appropriate controls, replication, and sample sizes. The conclusions must be drawn appropriately based on the data presented.

Reviewer #1: Partly

Reviewer #2: No

**Response:** the study was conducted scientifically with an appropriate methodology and the conclusion was drawn based on the findings.

3. Has the statistical analysis been performed appropriately and rigorously?
Reviewer #1: Yes

Reviewer #2: No

**Response:** this study used time to event data that is time to recovery therefore the statistical model was survival analysis using all necessary steps.

4. Have the authors made all data underlying the findings in their manuscript fully available?

The [PLOS Data policy](http://www.plosone.org/static/policies.action#sharing) requires authors to make all data underlying the findings described in their manuscript fully available without restriction, with rare exception (please refer to the Data Availability Statement in the manuscript PDF file). The data should be provided as part of the manuscript or its supporting information, or deposited to a public repository. For example, in addition to summary statistics, the data points behind means, medians and variance measures should be available. If there are restrictions on publicly sharing data—e.g. participant privacy or use of data from a third party—those must be specified.

Reviewer #1: Yes

Reviewer #2: Yes

5. Is the manuscript presented in an intelligible fashion and written in standard English?

PLOS ONE does not copyedit accepted manuscripts, so the language in submitted articles must be clear, correct, and unambiguous. Any typographical or grammatical errors should be corrected at revision, so please note any specific errors here.

Reviewer #1: No

Reviewer #2: No

**Response:** we have corrected English grammar, punctuation and spellings in the revised manuscript.

6. Review Comments to the Author

Please use the space provided to explain your answers to the questions above. You may also include additional comments for the author, including concerns about dual publication, research ethics, or publication ethics. (Please upload your review as an attachment if it exceeds 20,000 characters)

Reviewer #1: Thank you for inviting me to review the revision of this manuscript. In general, I find it to be much easier to read and the authors have taken most review comments under consideration. I have one major and minor comments related to this work.

Major comments

I am concerned about the outcome of “recovery”, which is highly subjective, being determined simply from clinician assessment. This is likely influences by a range of factors, including bed availability vs. demand, and may not have been consistent throughout the study period. This should be discussed and included as a limitation.
**Response:** the comment is addressed (p-19)

Minor comments:
Throughout the manuscript there is still clear evidence that the manuscript has not been edited by a native English speaker. Examples are given below, but after page 7 I stopped looking at grammar, as I believe this is beyond the scope of scientific review.

P3: “The hospital received 3293 children per year in average with the available 31 beds” change to “: The hospital admits an average of 3293 children per year, with 31 available beds”.
P3: “children with severe community acquired pneumonia were admitted in pediatric ward and further diagnose and treatment were provided by pediatricians, general practitioners and nurses” – change to “children with severe community acquired pneumonia
were admitted into the pediatric ward and further diagnosis and treatment were provided by
pediatricians, general practitioners and nurses”.
P5: “were admitted by sever community acquired pneumonia” – should be “admitted with severe CAP”
P5 – the medical charts were not extracted, presumably data were extracted from the medical charts.
P6 - “…inability to feed accompanying with SCAP” – change to “…inability to feed in addition to SCAP”
P7 – “fully vaccination was defined…” should be “full vaccination status was defined”
P7 – Very clumsy paragraph: “The patient records were first observed and an appropriate data extraction format was prepared in English. Data collectors used the data collection tool to collect the information from children’s charts. Charts were retrieved using the children’s registration number which was found in the database in the electronic system and the data clerks of the hospital were also support by identifying the charts”.
This can be rewritten much more clearly and simply, for example: “Patient records were retrieved using their unique registration number identified in the electronic hospital database. Charts and records were reviewed and data were extracted using a standardised English data extraction form”.

**Response:** we have corrected English grammar, punctuation and spellings in the revised manuscript based on the given recommendations.

Reviewer #2: Some changes have been made but inclusion criteria are still not clear. The signs for SCAP and danger signs should be given in tables of characteristics, even if grouped for analysis. The methods of analysis are still unclear.

In methods:
It would be helpful to include the definition of SCAP in the eligibility criteria rather than later under operational definitions. I am concerned that these essential features as well as individual danger signs and comorbidities do not appear in the baseline characteristics tables or analysis since they are likely to be major predictors of time to recovery.

**Response:** the definition of SCAP is included in the eligibility criteria p-4. The proportion of each danger sign and comorbidities were too low to describe in table of characteristics as well as in the analysis as an independent predictor. In addition there were also multiple responses. Therefore the signs for SCAP and danger signs are explained in text in the revised manuscript p-10.

I did not understand this section: “Predictor drug regimen violates the proportional hazard assumption and the Cox-Snell residual suggests that the Cox proportional hazard model (Cox regression) does not fit the data adequately. Therefore an extension of the Cox proportional hazard model is required for this data. Since predictors were tested as not time-dependent, the stratified Cox regression model was adequate to be used”. Was drug treatment time-dependent or not? If the model stratified by drug treatment then how is the hazards ratio for drug treatment calculated (I note the log rank test was highly significant)? The usual way to deal with this in common software packages is to declare the variable as a time-varying covariate (e.g. using the tvc() option with stcox in STATA, this does not require repeated measures).

In the next paragraph, what interaction was tested? Was it an interaction with time to deal with the violation of the proportional hazard assumption?

**Response:** the robust model of survival analysis is Cox regression (Cox proportional hazard). All predictors were checked for proportional hazard assumption using different methods. But Predictor drug regimen didn’t satisfy this assumption and it is not time-varying covariate as checked using “stcox i.drug , tvc( drug )” command in STATA. In addition model fitness for Cox regression was tested using Cox-Snell residual test but the data were not fitted. Therefore extension of the model was needed. Here stratified Cox regression is the extension (advanced forms of) Cox regression in survival analysis. The assumption of this model is non interaction assumption which has been checked to select either stratified Cox regression with interaction or without interaction. Based on this, stratified Cox regression without interaction was used. (P-8)

As drug regimen was stratification variable, hazards ratio was not calculated and it is explained in the limitation section. (P-19)

In results:
I agree with not examining risks for mortality. There will be some bias due to deaths and children leaving against advice if the 11% censored were more severely unwell for example. I should at least be mentioned as a limitation.
**Response:** the comment is addressed (p-19)

Should the heading “Incidence rate of SCAP” be “Incidence of recovery from SCAP”?

**Response:** corrected p-10

The terms “survival time of SCAP”, “survival time” and “The survival status of children with SCAP was estimated by...” are confusing (although possibly statistically correct) since recovery rather than survival is being studied. “Time to recovery” would be better.

**Response:** as stated in the operational definition survival time means time from admission to recovery that is the period stayed without recovery which is more informative since our focus is time to event that is survival.

In the discussion, case mix, severity and comorbidity differences are likely to underlie differences to other studies. Hence also the need to describe these for this study.
**Response:** the comments are addressed (p 18-19)

There is still a need to run a spelling and grammar check (e.g. “Anthrophometric”, “vaccins”, “ceftriaxon”, “”). There are also random capital letters: “In this study, Hyperactive airway disease...”, “...study conducted in Italy on the Prediction of delayed recovery from...” etc.

**Response:** we have corrected English grammar, punctuation and spellings in the revised manuscript.
