# Supplementary material for: Time to recovery and predictors of severe community-acquired pneumonia among pediatric patients in Debre Markos referral hospital, North West Ethiopia: A retrospective follow-up study
Source: PLoS One. 2020 Sep 25;15(9):e0239655. doi: 10.1371/journal.pone.0239655 (PMC7518609; doi:10.1371/journal.pone.0239655)
Supplement: Attachment — Submitted filename: Response to Reviewers.docx [file pone.0239655.s005.docx]

**Response to Reviewers**

We would like to thank you for your constructive comments and timely response. This is our response that addresses comments and suggestions of the reviewer and academic editor.

Reviewers' comments:

Reviewer's Responses to Questions

**Comments to the Author**

1. If the authors have adequately addressed your comments raised in a previous round of review and you feel that this manuscript is now acceptable for publication, you may indicate that here to bypass the “Comments to the Author” section, enter your conflict of interest statement in the “Confidential to Editor” section, and submit your "Accept" recommendation.

Reviewer #2: (No Response)

2. Is the manuscript technically sound, and do the data support the conclusions?

The manuscript must describe a technically sound piece of scientific research with data that supports the conclusions. Experiments must have been conducted rigorously, with appropriate controls, replication, and sample sizes. The conclusions must be drawn appropriately based on the data presented.

Reviewer #2: Partly

**Response: the conclusions were drawn based on the real findings.**

3. Has the statistical analysis been performed appropriately and rigorously?

Reviewer #2: No

**Response: statistical analyses were done appropriately and scientifically using all necessary steps and inferences were made rationally.**

4. Have the authors made all data underlying the findings in their manuscript fully available?

The [PLOS Data policy](http://www.plosone.org/static/policies.action#sharing) requires authors to make all data underlying the findings described in their manuscript fully available without restriction, with rare exception (please refer to the Data Availability Statement in the manuscript PDF file). The data should be provided as part of the manuscript or its supporting information, or deposited to a public repository. For example, in addition to summary statistics, the data points behind means, medians and variance measures should be available. If there are restrictions on publicly sharing data—e.g. participant privacy or use of data from a third party—those must be specified.

Reviewer #2: Yes

5. Is the manuscript presented in an intelligible fashion and written in standard English?

PLOS ONE does not copyedit accepted manuscripts, so the language in submitted articles must be clear, correct, and unambiguous. Any typographical or grammatical errors should be corrected at revision, so please note any specific errors here.

Reviewer #2: No

**Response: we have corrected English grammar, punctuation and spellings in the revised manuscript.**

6. Review Comments to the Author

Please use the space provided to explain your answers to the questions above. You may also include additional comments for the author, including concerns about dual publication, research ethics, or publication ethics. (Please upload your review as an attachment if it exceeds 20,000 characters)

Reviewer #2: There are improvements but the case definition characteristics ‘oxygen saturation <90% or central cyanosis or severe respiratory distress or inability to drink or breastfeed or vomiting everything, altered consciousness, and convulsions’ still need to be tabulated. Every child must have had at least one of these because they formed the case definition, so it is not clear why they are not analysed.

**Response:**

**Dear reviewer, the proportion of clinical presentations and co-morbidities are described in table of characteristics in the revised manuscript p-12. However, we didn’t use each danger sign as a predictor because there were multiple responses and some variables contain too small observations (<5%)**
In the discussion, case mix rather than ‘socio-economic differences in the study areas’ seems a more likely explanation for differences between studies.

**Response: corrected based on the recommendation p-18**

There are areas that still need language editing, for example:
P9: ‘308 (87.5%) fed breast for the first six months exclusively.’
P10: ‘313 (88.9%) children develop the event’
P17: ‘Stunted children recovered from the disease slower in compression to normal
children.’

**Response: the comments are addressed; P-11, 15, 19, and 20.**

7. PLOS authors have the option to publish the peer review history of their article ([what does this mean?](https://journals.plos.org/plosone/s/editorial-and-peer-review-process#loc-peer-review-history)). If published, this will include your full peer review and any attached files.


If you choose “no”, your identity will remain anonymous but your review may still be made public.


**Do you want your identity to be public for this peer review?** For information about this choice, including consent withdrawal, please see our [Privacy Policy](https://www.plos.org/privacy-policy).

Reviewer #2: No
