# Supplementary material for: Time to recovery and predictors of severe community-acquired pneumonia among pediatric patients in Debre Markos referral hospital, North West Ethiopia: A retrospective follow-up study
Source: PLoS One. 2020 Sep 25;15(9):e0239655. doi: 10.1371/journal.pone.0239655 (PMC7518609; doi:10.1371/journal.pone.0239655)
Supplement: Attachment — Submitted filename: Response to Reviewers.docx [file pone.0239655.s006.docx]

**Response to Reviewers**

We would like to thank you for your constructive comments and timely response. This is our response that addresses comments and suggestions of the reviewer and academic editor.

Additional Editor Comments (if provided):

Significant positive progress is evident and the manuscript is on the verge of acceptance. Reviewer 2 has offered a few pertinent recommended revisions for consideration, which hopefully will not take too much time or effort to consider for incorporation.


[Note: HTML markup is below. Please do not edit.]

Reviewers' comments:

Reviewer's Responses to Questions

**Comments to the Author**

1. If the authors have adequately addressed your comments raised in a previous round of review and you feel that this manuscript is now acceptable for publication, you may indicate that here to bypass the “Comments to the Author” section, enter your conflict of interest statement in the “Confidential to Editor” section, and submit your "Accept" recommendation.

Reviewer #1: All comments have been addressed

Reviewer #2: (No Response)

2. Is the manuscript technically sound, and do the data support the conclusions?

The manuscript must describe a technically sound piece of scientific research with data that supports the conclusions. Experiments must have been conducted rigorously, with appropriate controls, replication, and sample sizes. The conclusions must be drawn appropriately based on the data presented.

Reviewer #1: Yes

Reviewer #2: Yes

3. Has the statistical analysis been performed appropriately and rigorously?

Reviewer #1: Yes

Reviewer #2: Yes

4. Have the authors made all data underlying the findings in their manuscript fully available?

The [PLOS Data policy](http://www.plosone.org/static/policies.action#sharing) requires authors to make all data underlying the findings described in their manuscript fully available without restriction, with rare exception (please refer to the Data Availability Statement in the manuscript PDF file). The data should be provided as part of the manuscript or its supporting information, or deposited to a public repository. For example, in addition to summary statistics, the data points behind means, medians and variance measures should be available. If there are restrictions on publicly sharing data—e.g. participant privacy or use of data from a third party—those must be specified.

Reviewer #1: Yes

Reviewer #2: Yes

5. Is the manuscript presented in an intelligible fashion and written in standard English?

PLOS ONE does not copyedit accepted manuscripts, so the language in submitted articles must be clear, correct, and unambiguous. Any typographical or grammatical errors should be corrected at revision, so please note any specific errors here.

Reviewer #1: Yes

Reviewer #2: Yes

6. Review Comments to the Author

Please use the space provided to explain your answers to the questions above. You may also include additional comments for the author, including concerns about dual publication, research ethics, or publication ethics. (Please upload your review as an attachment if it exceeds 20,000 characters)

Reviewer #1: I have no further comments, authors responded satisfactorily to a previous review.

Reviewer #2: The authors have made extensive revisions which have improved the manuscript. Defining SCAP and treatment failure is very helpful. The GLM model is an improvement and the signs associated with failure at 48h (convulsions, cyanosis, hypoxia, HIV, MAM & SAM) appear to be appropriate and in line with prior studies.

There are a few areas that could be further improved:

Major comment: in figure 1, please indicate deaths before 48h separately. This is also needed in Results at around line 202 since it is the most important outcome.
**Response: dear reviewer, the outcome of this study was recovery not death so that we can’t indicate death in the Kaplan-Meier survival curve (figure), our data is time to recovery data not time to death.**

Please mention in the methods that children who immediately started second-line antibiotics were excluded (as per the response to reviewers) and give the number in results.

**Response: dear reviewer, we haven’t excluded children from the study based on the treatments given.**
In figure 1, exclusions can be simply described as “Not meeting SCAP criteria or out of the study age range”.

**Response: the inclusion criteria in the method section already indicated that the study participants were SCAP patients from 1 month to 15 years only. This means patients who did not meet SCAP criteria or out of the study age range were excluded**.
At line 178, please move “Respiratory distress in this study context was considered as the presence of grunting and head nodding” and “In this study, an acute form of malnutrition was assessed among the participants using the WHO Z-score charts of weight for height or length. Z-scores of between -3SD and ≤ -2SD were equated to moderate acute malnutrition and Z-scores of ≤ -3SD were equated to severe acute malnutrition, also the presence of kwashiorkor was considered as severe acute malnutrition.” to the Methods section.

**Response: comments are addressed based on the recommendations, page 6-8.**

It is important to note that previous studies may have used the WHO 2005 pocketbook definition of severe pneumonia which is much less severe than the 2013 version (2013 version is equivalent to the 2005 ‘very severe pneumonia’ syndrome). This applies to “studies done in Kenya (10) and South Africa (14)” at line 238. In the Kenyan study, ‘very severe pneumonia’ had a failure rate of around 30%. I am not sure if any of the South African children would have met the 2013 severe criteria since only amoxicillin treatment was given.
Please comment on the frequency of x-ray findings in comparison with other studies such as PERCH.
Please check spellings and spaced throughout e.g. “Amipicilin” at line 237, “...gram-negative bacteria are resistant to ampicillin and gentamycin...” at line 272 should be “...Gram-negative bacteria are resistant to ampicillin and gentamicin...”. Space is missing at lines 114 “6hourly” and 210-211 “p value<0.001”, please check for others.

**Response: comments are addressed page 10, 12, 14, 16, 18, 20.**

7. PLOS authors have the option to publish the peer review history of their article ([what does this mean?](https://journals.plos.org/plosone/s/editorial-and-peer-review-process#loc-peer-review-history)). If published, this will include your full peer review and any attached files.

If you choose “no”, your identity will remain anonymous but your review may still be made public.


Do you want your identity to be public for this peer review? For information about this choice, including consent withdrawal, please see our [Privacy Policy](https://www.plos.org/privacy-policy).

Reviewer #1: No

Reviewer #2: **Yes:** James A Berkley
